# Correction: Enhanced luminescence and tunable magnetic properties of lanthanide coordination polymers based on fluorine substitution and phenanthroline ligand

**DOI:** 10.1039/c9ra90044f

**Published:** 2019-06-11

**Authors:** Xun Feng, Yapei Shang, Heng Zhang, Rongfang Li, Weizhou Wang, Daoming Zhang, Liya Wang, Zhongjun Li

**Affiliations:** College of Chemistry and Chemical Engineering, Henan Key Laboratory of Function Oriented Porous Materials, Luoyang Normal University Luoyang 471934 China fengx@lynu.edu.cn; School of Life Science and Technology, Nanyang Normal University Nanyang 473601 China wlya@lynu.edu.cn; College of Chemistry and Molecular Engineering, Zhengzhou University Zhengzhou 450001 China

## Abstract

Correction for ‘Enhanced luminescence and tunable magnetic properties of lanthanide coordination polymers based on fluorine substitution and phenanthroline ligand’ by Xun Feng *et al.*, *RSC Adv.*, 2019, **9**, 16328–16338.

The authors regret that the funding information in the acknowledgements of the original article was incorrect. Natural Science Foundation of China (No. 21671114 and U1804113), should be “Natural Science Foundation of China (No. 21671114 and U1804131)”.

The Royal Society of Chemistry apologises for these errors and any consequent inconvenience to authors and readers.

## Supplementary Material

